# Incidence and predictors of mother-to-child transmission of HIV among HIV-exposed infants in public General hospitals of Tigray, Ethiopia, 2020: Retrospective cohort study

**DOI:** 10.1371/journal.pone.0358878

**Published:** 2026-10-22

**Authors:** Gebrezgiher Gebresselassie Gerezgiher, Tesfay Gebreslassie, Guesh Teklu Woldemariam, Yohannes Ashebir Tesfamicale, Haftom Gebrehiwot Misgna, Degena Bahrey Tadesse, Kidane Zereabruk, Naomi Lijalem, Willi Bahre

**Affiliations:** 1 Department of Nursing, College of Medicine and Health Science, Adigrat University, Adigrat, Ethiopia; 2 Department of Pediatric and Child Health Nursing, School of Nursing, College of Health Science, Mekelle University, Mekelle, Ethiopia; 3 Department of Adult Health Nursing, School of Nursing, College of Health Science, Aksum University, Aksum, Ethiopia; University of Rwanda College of Medicine and Health Sciences, RWANDA

## Abstract

**Background:**

Mother-to-child transmission (MTCT) of human immunodeficiency virus (HIV) remains a major public health challenge, particularly in low-income countries. Previous studies conducted in Ethiopia were largely limited to single health facilities and did not adequately assess important predictors, including maternal viral load, paternal enrollment in antiretroviral therapy (ART), and breastfeeding duration. Therefore, this study aimed to determine the incidence and predictors of MTCT of HIV among HIV-exposed infants in public general hospitals of Tigray, Ethiopia**.**

**Methods:**

Hospital-based retrospective cohort study on 485 randomly selected HIV exposed infants attending in public General Hospitals was conducted. All HIV exposed infants attending in public general hospitals of Tigray were the source of population. Data were entered into Epidata version 4.6.1 and exported to SPSS version 23 for analysis. Binary logistic regression model was used and variables at p-value < 0.25 in bivariate analysis were entered into a multivariable logistic regression analysis to examine the predictors of mother-to-child transmission of HIV. Level of significance was declared at p-value <0.05.

**Result:**

A total of 444(91.5%) records were included in the analysis. At the end of follow up, 17 (3.8%) (95% CI: 2.3%–5.8%) of the HIV exposed infants became HIV positive. Poor infant adherence (AOR: 5.6; 95% CI: 1.02–30.2), father not enrolled to ART (AOR: 4.4; 95% CI: 1.2–16.7), age of infant at enrolment >6 weeks (AOR: 4.4; 95% CI: 1.2–17.0), mothers enrolled to PMTCT during labor and delivery or after (AOR: 7.9; 95% CI: 1.4–44.7) and mothers on advanced WHO clinical stage at base line(AOR: 4.2; 95% CI: 1.02–17.89) were significantly associated with mother-to-child transmission of HIV.

**Conclusion:**

In this study MTCT of HIV is still high. Poor adherence of infant for PMTCT, HIV positive father not enrolled to ART, infant age > 6 weeks at enrollment, mothers enrolled to PMTCT during labor and delivery or after and mothers on advanced WHO clinical stage were predictors of mother to child transmission of HIV.

## Background

Human immunodeficiency virus (HIV) is from a special family of viruses known as retroviruses and it was first discovered in 1981 [1]. Pediatric acquired immunodeficiency syndrome (AIDS) was first described after 18 months after the first cases were reported in adults in 1982 [2].

Mother-to-child transmission (MTCT) of HIV remains the primary route through which children acquire the infection. It refers to the transmission of HIV from an HIV positive mother to her child during pregnancy, childbirth, or breastfeeding, accounting for approximately 90% of HIV infections in children [3–5]. Infants born to mothers living with HIV are considered HIV-exposed and may acquire the infection at any of these stages if appropriate preventive measures are not implemented [1,4,5].

In the absence of preventive interventions rates of MTCT of HIV ranges between 20–45% in which 5% – 10% during pregnancy, 10% – 15% during labor and delivery, and 5% – 20% during breastfeeding [5]. However, the use of antiretroviral (ARV) medications for both the mother and the infant has been shown to significantly reduce this risk, particularly when initiated early and maintained throughout pregnancy and breastfeeding [4].

Despite advances in prevention strategies, pediatric HIV continues to be a major global public health concern, especially in low-income countries where access to healthcare services may be limited [6]. In 2018, of the total of 1.7 million new HIV infection globally around 9.4% are 0–14 years of children in which 90% of them are infected due to MTCT during pregnancy, labor and delivery and breastfeeding period [1,4,7,8].

Africa remains the region most heavily impacted by HIV, where the rate of mother-to-child transmission (MTCT) is estimated at 18% [9]. In 2017, out of 180,000 newly reported HIV infections among children worldwide, 70% occurred in 21 high-burden countries, including Ethiopia [10]. Within these countries, the MTCT rate varies considerably, ranging from a high of 26% in Indonesia, Angola, and Nigeria to a low of 5% in South Africa and Botswana [10].

Ethiopia is among the 21 high-burden countries, with an overall HIV prevalence of 0.96%. This prevalence shows marked regional variation, from less than 0.1% in the Somali region to 4.8% in the Gambella region [1,11]. Consistent with this regional variation, studies conducted in Ethiopia have reported that the rate of mother-to-child transmission (MTCT) of HIV ranges from 2.1% to 15.7% [12–16].

Evidence indicates that multiple factors influence MTCT of HIV. These include poor adherence to antiretroviral therapy, insufficient antenatal care visits, home delivery, failure of infants to receive antiretroviral (ARV) prophylaxis at birth, low maternal CD4 count, breast complications during breastfeeding, and lack of enrollment in prevention of mother-to-child transmission (PMTCT) programs, as documented in various studies [17–21].

An initiative known as the “Global Plan towards the Elimination of New HIV Infections among Children by 2015 and Keeping Their Mothers Alive” was introduced in July 2011 with the goal of eliminating new HIV infections among children and ensuring the survival of their mothers [22]. This initiative significantly reduced new pediatric HIV infections by 60% in the 21 high-burden countries of sub-Saharan Africa. Nevertheless, MTCT of HIV continues to contribute substantially to new infections among children [10].

Despite ongoing efforts by governmental and non-governmental organizations to retain HIV-exposed infants in care throughout the exposure period, approximately 16% of HIV-exposed infants globally were infected with HIV in 2012 [23]. Studies conducted in Ethiopia further reveal that the MTCT rate at 18 months remains higher than the national target set by the Health Sector Transformation Plan, which aimed to achieve 0% transmission by 2020 [12,14,21].

Previous studies conducted in different parts of Ethiopia have investigated the incidence and determinants of mother-to-child transmission (MTCT) of HIV. However, many of these studies were based on data from a single health facility and included relatively small sample sizes. In addition, their findings were not always consistent, and several important variables, including maternal viral load, paternal enrollment in antiretroviral therapy (ART), and breastfeeding duration, were not adequately assessed. Moreover, as guidelines for lifelong HIV treatment and MTCT prevention are periodically updated, there is a continued need for research reflecting these changes.

This study addresses important variables that were not sufficiently addressed in previous researches, including maternal viral load, duration of breastfeeding, and paternal enrollment in ART. Therefore, the primary objective of this study is to determine the incidence and predictors of MTCT of HIV among HIV-exposed infants in selected public general hospitals of Tigray, Ethiopia, in 2020.

## Methods

### Study setting and period

The study was conducted in public General hospitals of Tigray providing Prevention of Mother-to-Child Transmission service from February – March 2020. Tigray region is one of the nine regional states of Ethiopia located on the northern part of the country bordered by Eritrea to the north, Sudan to the west, the Afar Region to the east, and the Amhara region to the south and southwest. It spread across 54,572.6 sq, km administratively divided into seven Zones including one special Zone, Mekelle. According to CSA, 2007 population census projected the region has an estimated total population of 5,541,736 with sex composition of 49% male and 51% female in 2019 [24]. The region has two specialized hospitals, 14 general hospitals, 22 primary hospitals, 202 health centers and 712 health posts, of these all hospitals and health centers are providing ART and PMTCT services [25].

Three-year duration of HIV exposed infants and their mothers charts registered from January 2016 to December 2018 were included. According to the registration records of HIV-exposed infants at the selected general hospitals, a total of 812 HIV-exposed infants were enrolled in follow-up care during the three-year study period.

### Study design

Hospital-based retrospective cohort study design was used

### Source of population

All HIV-exposed infants enrolled in PMTCT services in all public General Hospitals of Tigray region who registered from January 2016 to December 2018.

### Study population

All selected HIV-exposed infants in selected public General Hospitals of Tigray

### Eligibility criteria

#### Inclusion criteria.

All HIV exposed infants who had documented HIV test results either DNA-PCR before 18 months of age or rapid antibody testing after six weeks of cessation of breastfeeding between January 2016 and December 2018 in the selected hospitals were included.

#### Exclusion criteria.

HIV exposed infants enrolled to PMTCT service from January 2016 to December 2018 in the selected Public General hospitals of Tigray, not confirmed or registered their HIV test result.

#### Sample size determination.

For the first objective single proportion formula was made by considering Mother to child transmission rate 15.7% from previous study [14].


$$\begin{array}{c} \text{n}=\frac{{\left(\text{Z}\frac{\alpha }{\text{2}}\right)}^{\text{2}}*\text{p}(\text{1}-\text{p})}{{\text{d}}^{\text{2}}} \end{array}$$

Where: n = the required sample size, Z = the standard score corresponding to 95% CI,

P = the proportion of MTCT of HIV, d2 = level of precision (margin of error)


$$\begin{array}{c} \text{n}=\frac{{\left(\text{1}.\text{96}\right)}^{\text{2}}*\text{0}.\text{157}*(\text{1}-\text{0}.\text{157})}{{\left(\text{0}.\text{05}\right)}^{\text{2}}}=\text{203} \end{array}$$

Then after adding 10% contingency rate for an incomplete chart and 1.5 design effect, the final sample size was, n = 336

For the second objective using double proportion formula sample size was calculated using Epi Info Version 7 Stat Calc by the assumption of 95% CI and 80% power with exposed to unexposed ratio 1:1 and using 1.5 design effect, by considering variables like, infant feeding practice, infant age at enrolment to PMTCT, residence and ANC follow up as the major predictor variables. Moreover, infant feeding practice was considered as the independent predictor since it gives the maximum sample size (to reduce role of chance), so n = 485 (Table 1).

**Table 1:** Sample size calculation for incidence and predictors of mother to child transmission of HIV among HIV exposed infants in general hospitals of Tigray, Ethiopia 2020.

Variables	Assumption	Sample size by adding 10% contingency and 1.5 design effect	Reference
Infant feeding practice	OR= 3.68, P1 = 14.8, P2 = 4.5	294 + 29 = 323*1.5 = 485	[13]
Infant age at enrolment	OR=4.78, P1 = 23.07, P2 = 5.9	152 + 15 = 167*1.5 = 251	[18]
Residence	OR=3.8, P1 = 36.6, P2 = 13.2	122 + 12 = 134*1.5 = 201	[14]
ANC follow up	OR=5.95 P1 = 21.1, P2 = 4.3	144 + 14 = 237	[21]

Where p is probability of the outcome, OR is odds ratio.

### Sampling techniques and sampling procedure

First the Public general hospitals of Tigray providing PMTCT service were listed, then from all 14 Public general hospitals in Tigray region providing PMTCT service 40% of them (six general hospitals) were selected by using lottery method. The selected general hospitals that were included in the study were; Lemelem Karl, Mekelle, Adwa, St.Mery (Aksum), Suhul and Kahsay Abera general hospital. Then sample size for each selected general hospitals was proportionally allocated according to the number of HIV-exposed infants registered for follow up from January 2016 to December 2018. After that, medical records of exposed infants were selected by using simple random sampling from the registration book of exposed infants follow up of the selected hospitals.

### Study variables

#### Dependent variable.

MTCT of HIV

#### Independent variables.

**Socio-demographic characteristics of the mother:** Age at delivery, marital status of mother, Residence, Occupation, Maternal educational status and religion

**Infant factors: Sex,** infant feeding practice, birth Weight, ARV prophylaxis at birth, Age at enrolment to PMTCT, growth pattern, ARV prophylaxis adherence, Use of CPT, CPT prophylaxis adherence, duration of breast feeding,

**Paternal Factors:** Father HIV status and father enrollment in antiretroviral therapy (ART)

**Clinical factors of the mother:** WHO clinical Stage, CD4 count during pregnancy, viral load during pregnancy, time of knowing HIV serostatus, PMTCT intervention, ART Adherence, nutritional status, breast condition during breast feeding, Syphilis test result during pregnancy, time of ART initiation, disclosure status of the mother, opportunistic infections at base line and functional status at base line

**Obstetric factors:** ANC follow-up, Place of delivery, Mode of delivery, Gravidity, Parity, Postnatal follow up

### Data collection tools and procedures

A structured data extraction form was developed by adapting relevant variables from the HIV-exposed infant follow-up card, the adult HIV care and treatment follow-up/intake forms, the Ethiopian national HIV care and treatment package, and previously published studies [1,5,20,26]. Data was collected via review of HIV-exposed infant and mothers’ medical records. Detailed information on the records and management of the infants was obtained from key informants (health care providers) working at the PMTCT unit of the selected General Hospitals. First, data was collected from infant’s registration cards and then from the infant’s mother cards. Data was collected by four trained diploma nurses working out of the selected hospitals and supervised by two BSc nurses who are working on ART out of the selected hospitals.

### Data quality control

The data extraction tool was developed based on integrated PMTCT register, exposed infant follow-up registration book, medical record and after extensive review of related journal articles. Experienced data collectors and supervisors were recruited and training was given for two days on how to collect data, general objective, relevance of the study, and confidentiality of information. Close supervision was made and filled formats and checklist was checked on a daily basis for completeness and consistency. Additionally 5% of pretest of the tool was done out of the selected area in Wukro general Hospital before starting of data collection. Based on the pretest findings, the data extraction form was refined by clarifying variable definitions and coding instructions, reorganizing the sequence of variables to improve the flow of data extraction before both the data collectors and supervisors go to the field. Data was cross-checked, validated and all the discrepancies were resolved.

### Data analysis procedure

Data was coded and entered into Epi data version 4.6.1 and was exported to SPSS version 23 for cleaning and analysis. Cleaning of data was made to check for any inconsistencies, errors in coding, missing values, out of range values, unexpected data or outliers and inconsistencies were cross checked with the data in hard copy and necessary correction measures were taken. Descriptive statistics was presented by frequency tables, texts, mean, median and figures. HIV serostatus of the HIV-exposed infants at the age of 18 months or the last result after six weeks of cessation of breast feeding was used to compute the incidence of MTCT of HIV among HIV exposed infants. Multicollinearity was checked using variance inflation factor to assess the relationship of the independent variables between themselves (VIF = 1.03) and Model fitness was also checked using Hosmer and Lemeshow goodness of fit and was fitted (0.439). Bivariate and Multivariable logistic regression analysis with a 95% confidence interval was employed to assess the association between the independent variables and the outcome variable. Variables at p-value less than 0.25 in bivariate analysis were entered into a multivariable logistic regression analysis to examine the main predictors of mother-to-child transmission of HIV. Variables persisted to be associated with the outcome at P < 0.05 were used to declare whether association between outcome and independent variables existed.

### Operational definitions

**Mother-to-child transmission (MTCT) of HIV:** An HIV-exposed infant who was confirmed to be HIV-positive during the follow-up period, either by a positive HIV DNA PCR test performed at 6 weeks or 6 months of age, or by a positive HIV antibody test at 18 months of age or at least 6 weeks after cessation of breastfeeding, in accordance with the national HIV testing guidelines [1].

**HIV Exposed Infants:** Infants born from mothers with positive serostatus of HIV [1].

**HIV Serostatus of infant:** HIV test result identified with DNA/PCR prior to 18 months of age, or by rapid antibody test at 18 months of age and 6 weeks of cessation of breast feeding [20].

**Growth pattern:** Rate as normal, if the HIV exposed infant had no any evidence of growth failure, or termed as growth failure if weight for age is < 3 percentile [5]

**Breast disease during breast feeding:** If the mother had any of these cracked nipples, mastitis, or breast abscess [5].

**Nutritional Status of The Mother:** Measured using mid upper Arm Circumference (MUAC): If ≥ 23 cm = Not malnourished, < 23 cm = Malnourished [20]

**ART Adherence:** Measured based on number of missed doses within 60 days. Equal to or greater than 95% adherence, i.e., missing only 1 out of 30 doses or missing 2 from the 60 doses, 85–94% adherence, i.e., missing 2–4 doses out of 30 doses or 4–9 doses from 60 doses, less than 85% adherence, i.e., missing _ > 5 doses out of 30 doses or > 10 doses from 60 doses rated as good, fair and poor respectively [1].

### Ethics approval and consent

Ethical clearance letter was obtained from institutional Review Board (IRB) of College of Health Sciences, Mekelle University ((**ERC 1524/2020)**. Subsequent letter of permission was also obtained from Tigray regional health bureau and official permission was obtained from respective medical director of selected general hospitals. Confidentiality was assured for all the information provided. The information collected from the medical records of the infant and their mothers was kept confidential and was used only for the study purpose and the names of the participants were not included in the abstracted data. As this was a retrospective study using secondary data obtained from patient cards which is available in the Hospital, informed consent from participants was not feasible. All methods were performed in accordance with declaration of Helsinki.

## Results

### Socio-demographic characteristics of mothers of HIV exposed infants

A total of 485 HIV exposed infants along with their mothers who were enrolled in PMTCT care enrolled in PMTCT care in Public General Hospitals of Tigray Regional State from January 2016 to December 2018 were reviewed. Of these, 444 (91.5%) mother–infant pairs were eligible and included in the final analysis. The data was collected from six General Hospitals. Two hundred eighty-seven (64.6%) of mothers were in the age group of 25–34 years with the mean and SD of 30.81 (± 4.97) years. Two hundred sixty (60.8%) of the mothers were married. Regarding educational status 229 (51.6%) were primary and 52(11.9%) were not educated, Two hundred thirty-four (52.7%) of the mothers were employed. Three hundred eighty-seven (87.2%) of them were Orthodox Christians (Table 2).

**Table 2:** Baseline socio-demographic characteristics of the mothers of HIV exposed infants in general hospitals of Tigray, Ethiopia, 2020.

Variable (n = 444)	Category	Frequency (%)	HIV test result of infant	
			Positive (%)	Negative (%)
Age of mother	15-24	41(9.2)	0	41(100)
	25-34	287(64.6)	12 (4.2)	275 (95.8)
	>= 35	116(26.2)	5 (4.3)	111 (95.7)
Residence	Urban	342(77)	11 (3.2)	331 (96.8)
	Rural	102(23)	6 (5.9)	96 (94.1)
Occupation	Employed	234(52.7)	6 (2.6)	228 (97.4)
	Unemployed	210(47.3)	11 (5.2)	199 (94.8)
Religion	Orthodox	387(87.2)	15 (3.9)	372 (96.1)
	Muslim	55(12.4)	2 (3.6)	53 (96.)
	Other	2(0.4)	0	2 (100)
Marital status	Married	270(60.8)	7 (2.6)	263 (97.4)
	Single	91(20.5)	4 (4.4)	87 (95.6)
	Divorced/Widowed	83(18.7)	6 (7.2)	77(92.8)
Educational status	Not educated	53(11.9)	4(7.5)	49 (92.5)
	Primary	229(51.6)	9 (3.9)	220 (96.1)
	Secondary and above	162(36.48)	4 (2.5)	158(97.5)

* Other protestant.

### Clinical characteristics of the mother

Three-fourths, 333(75%) of the mothers were working at baseline. Three hundred three (68.2%) of them knew their HIV status before they became pregnant for the index pregnancy. More than two-third, 315(70.9%) of the mothers take CPT prophylaxis. Three hundred forty-one (76.8%) of the mothers had good ART adherence during pregnancy, and one hundred ninety-two (43.2%) of them had 200–350 cells/mm3 CD4 count at baseline with a median of 315 and interquartile range of 203. During pregnancy, 179 (40.3%) of the mothers had >=500 cells/mm3 with a median of 458 and an interquartile range of 287 respectively and 432(97.3%) of them were on mild WHO clinical stage during the pregnancy (Table 3).

**Table 3:** Clinical characteristics of mothers of HIV exposed infants in Public General Hospitals of Tigray, Ethiopia, 2020.

Variable (n = 444)	Category	Frequency (%)	HIV test result of infant	
			Positive(%)	Negative(%)
WHO clinical stage at last visit of pregnancy	Mild stage(I,II)	432(97.3)	9 (2.1)	423 (97.9)
	Advanced stage(III, IV)	12(2.7)	8 (66.7)	4 (33.3)
CD4 count at baseline	>=500	42(9.5)	0 (0)	42 (100)
	351-499	115(25.9)	0(0)	115 (100)
	200-350	192(43.2)	4 (2.1)	188 (97.9)
	<200	95(21.4)	13 (13.7)	82 (86.3)
Last CD4 count during pregnancy	>=500	179(40.3)	1 (0.6)	178 (99.4)
	351-499	153(34.5)	3(2)	150(98)
	200-350	89(20.0)	3 (3.4)	86 (96.6)
	<200	23(5.2)	10(43.5)	13 (56.5)
Functional status at baseline	Working	333(75.0)	8 (2.4)	325 (97.6)
	Ambulatory	99(22.3)	4 (4)	95 (96)
	Bedridden	12(2.7)	5 (41.7)	7 (58.3)
Time of HIV diagnosis	Before pregnancy	303(68.2)	11 (3.6)	292 (96.4)
	During pregnancy or after	141(31.8)	6(4.3)	135(95.7)
Enrolment to PMTCT	Yes	442(99.5)	17(3.8)	425 (96.2)
	No	2(0.5)	0	2(100)
ART adherence at last visit of pregnancy	Good	341(76.8)	9(2.6)	332(97.4)
	Fair	72(16.2)	4(5.6)	68(94.4)
	Poor	31(7.0)	4(12.9)	27(87.1)
OIs at base line	No	175(39.4)	2(1.1)	173(98.9)
	Yes	269(60.6)	15(5.6)	254(94.4)
Types of OIs	42(15.6%)= TB, 67(24.8%)=chronic diarrhea,117(43.3%)= mucocutanous manifestations 74(28.4%) =recurrent URTIs			
Disclosure status	Yes	334(75.2)	8(2.4)	326(97.6)
	No	110(24.8)	9 (8.2)	101 (91.8)
Last viral load during pregnancy	Undetectable	356(80.2)	10 (2.8)	346(97.2)
	Detectable	88(19.8)	7 (8)	81 (92)
Nutritional status during pregnancy	Not malnourished	353(79.5)	9(2.5)	344(97.5)
	Malnourished	91(20.5)	8(8.8)	83(91.2)

Two hundred sixty-nine (60.6%) mothers had at least one opportunistic infection at baseline, of those with OIs, 117(43.5%), 74 (27.5%) had mucocutaneous manifestations and recurrent upper respiratory tract infections respectively. None of the mothers had breast disease during breastfeeding. Three-fourths of the mothers, 334 (75.2%) had disclosed their HIV status for others. Of these disclosed their HIV status 267(60.1%) of them were disclosed for their husband. Three hundred fifty-six (80.2%) of the mothers had undetectable viral load during pregnancy. Three hundred fifty-three (79.5%) of the mothers had no malnutrition during pregnancy. A syphilis test result was negative among 432 (97.3%) mothers during pregnancy (Table 3).

Regarding the enrollment of the mothers to PMTCT, 442(99.5%) of the mothers were enrolled to PMTCT, of these enrolled to PMTCT, 270 (60.9%) of them were on HAART before (Fig 1).

**Fig 1:**
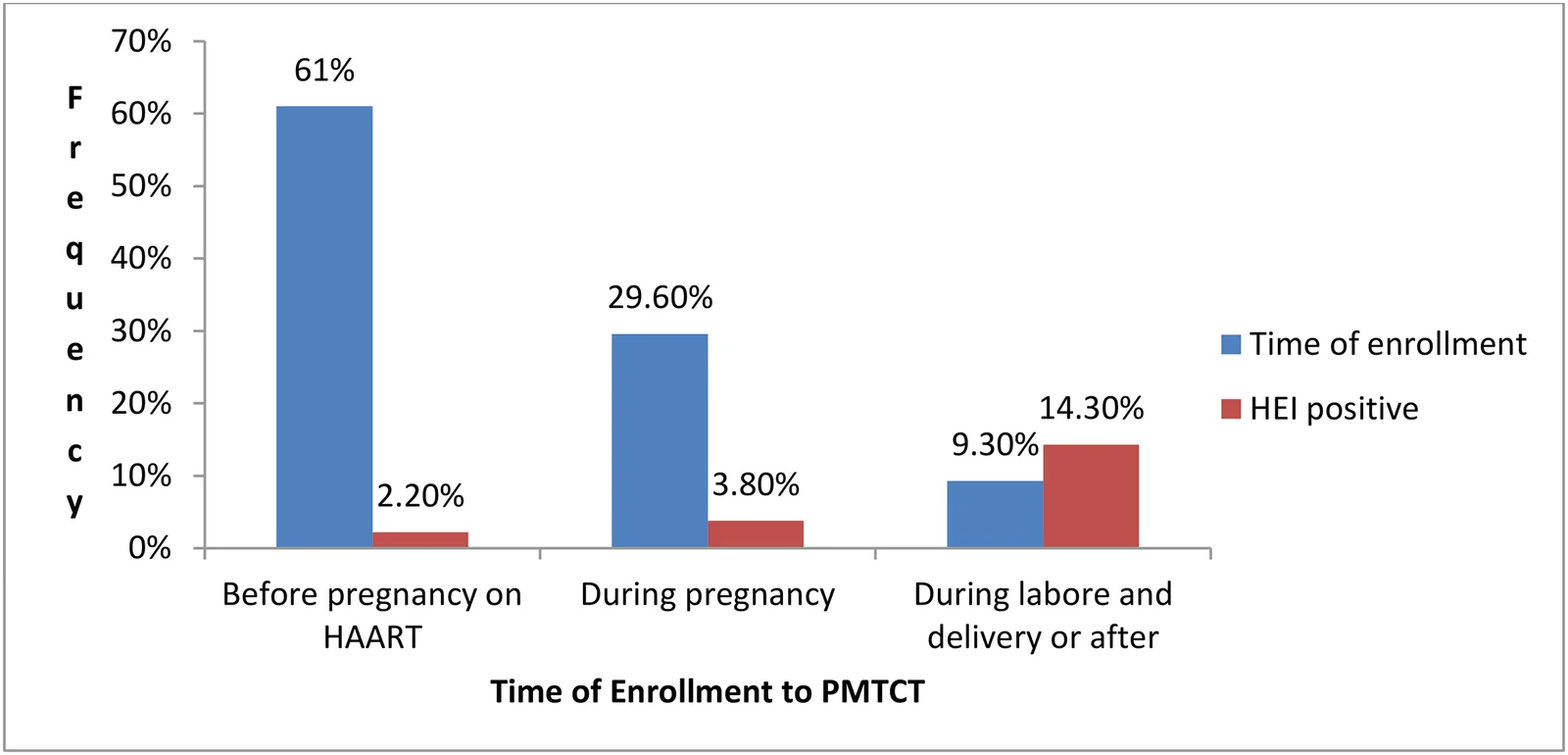
Time of HIV-positive mothers’ enrollment for pmTCT in public general hospitals of Tigray, Ethiopia, 2020.

Regarding the WHO clinical staging of the mothers at base line, three hundred fifty-six (80.2%) of them were on mild stage and 10.2% of the HEI whose mothers were on advanced WHO clinical stage at base line were HIV positive (Fig 2).

**Fig 2:**
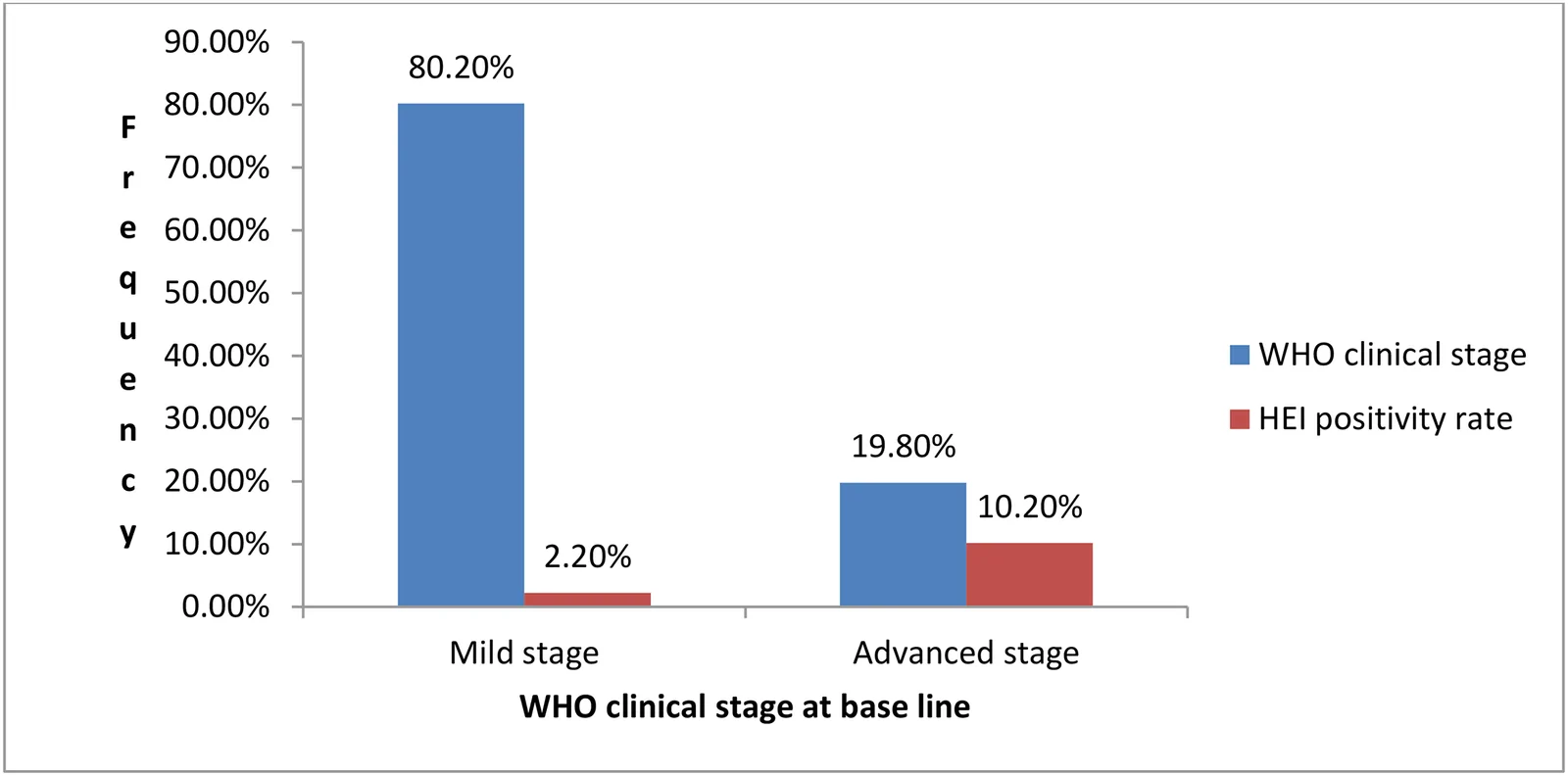
WHO clinical staging of mothers with HIV-exposed infants at baseline in Tigray, Ethiopia, 2020.

### Obstetrics characteristics of the mother

The obstetric history of mothers was also collected. Accordingly, 204(45.9%) of the mothers were gravida three. Four hundred fifteen (93.5%) of them hade ANC follow up during pregnancy of the index pregnancy, of these 281(63.3%) hade >=4 ANC visits. Regarding the place of delivery, four hundred thirty-two (97.3%) of the mothers had delivered at Health institution, and 364 (82%) of them were delivered through SVD. Two hundred eighty-three (63.7%) of the mothers hade postnatal follow up after delivery of the index pregnancy (Table 4).

**Table 4:** Obstetric characteristics and HIV exposed infant characteristics in General Hospitals of Tigray, Ethiopia 2020.

Variables	Category	Frequency(%)	Last HIV test result of infant	
			Positive(%)	Negative(%)
ANC follow up	Yes	415(93.5)	14(3.4)	401(96.6)
	No	29(6.5)	3(10.3)	26(89.7)
Number of ANC visit(n = 415)	>=4	281(67.7)	2(0.7)	279(99.3)
	2-3	130(31.3)	10(7.7)	120(92.3)
	1	4(0.96)	2(50)	2(50)
Postnatal follow up	Yes	283(63.7)	10(3.5)	273 (96.5)
	No	161(36.3)	7(4.3)	154 (95.7)
Gravidity	I	71(16.0)	2(2.8)	69 (97.2)
	II	169(38.0)	6(3.6)	163(96.4)
	III and above	204(46.0)	9(4.4)	195(95.6)
Number of children(parity)	1	90(20.3)	2(2.2)	88 (97.8)
	2	169(38.0)	10(5.9)	159 (94.1)
	>=3	185(41.7)	5(2.7)	180 (97.3)
Place of delivery	Health institution	432(97.3)	14(3.2)	418 (96.8)
	Home	12(2.7)	3(25)	9 (75)
Mode of delivery	SVD	364(82.0)	15(4.1)	349 (95.9)
	CS	80(18.0)	2(2.5)	78(97.5)
Sex of infant	Male	237(53.4)	9(3.8)	228(96.2)
	Female	207(46.6)	8(3.9)	199(96.1)
Birth weight in KG	>=2.5	410(92.3)	14(3.4)	396(96.6)
	<2.5	34(7.7)	3(8.8)	31(91.2)
Growth pattern	Normal	398(89.6)	4(1)	394(99)
	Growth failure	46(10.4)	13(28.3)	33(71.7)
Feeding practice before six months age	EBF	433(97.5)	16(3.7)	417(96.3)
	ERF	11(2.5)	1 (9.1%)	10 (90.9)
	Mixed feeding	0(0)	0	0
Feeding practice after six months	BF + CF	419(94.4)	15(3.6)	404 (96.4)
	Stop BF	25(5.6)	2 (8)	23(92)
Duration of BF in weeks	<= 48 weeks	208(46.8)	6(2.9)	202(97.1)
	>48 weeks	236(53.2)	11(4.7)	225(95.3)
ARV prophylaxis at birth for six weeks	Yes	418(94.1)	4(1)	414(99)
	No	26(5.9)	13(50)	13(50)
Age in weeks at enrolment to PMTCT	<=6	362(81.5)	7 (1.9)	355 (98.1)
	>6	82(18.5)	10 (12.2)	72(87.8)
Father’s ART enrollment(n = 320)	Enrolled	272(85)	8(2.9)	264(97.1)
	Not enrolled	48(15)	8(16.7)	40(83.3)

### Characteristics of HIV exposed infants and incidence of MTCT of HIV

At the end of follow-up, 17 (3.8%) of HIV-exposed infants were confirmed to be HIV-positive (95% CI: 2.3%–5.8%). Two hundred thirty-seven (53.4%) of the HIV exposed infants were male. Birth weight of 410(92.3%) HIV exposed infants was >=2.5 kg with a median of 3.00 kg and an interquartile range of 0.5. Three hundred sixty-two (81.5%) of them were enrolled in PMTCT at their age of <= 6 weeks and the mean age in weeks of enrollment was 6.64 and SD ± 2.183 respectively.

The growth pattern of 398 (89.6%) infants was normal. Four hundred thirty-three (96.4%) infants were on exclusive breastfeeding and none of them were on mixed feeding until 6 months of age.

After six months of age, 419(94.4%) of them were on breastfeeding +complementary feeding; only 5.6% of them were weaned off at six months of age. The median duration of breastfeeding in weeks was 52 and interquartile range of 12. Three hundred twenty (72.1%) of fathers of them were HIV positive, and 96(21.6%) of them were unknown. Of those HIV positive fathers, 272(85%) of them were enrolled in ART (Table 4).

Regarding adherence of HIV exposed infants for PMTCT, three forth 336(75.7) of them had good adherence, of those with good adherence 7(2.1%) of them were positive for HIV and of 26(5.9%) of HEIs with poor adherence 5(19.2%) of them were positive for HIV (Fig 3).

**Fig 3:**
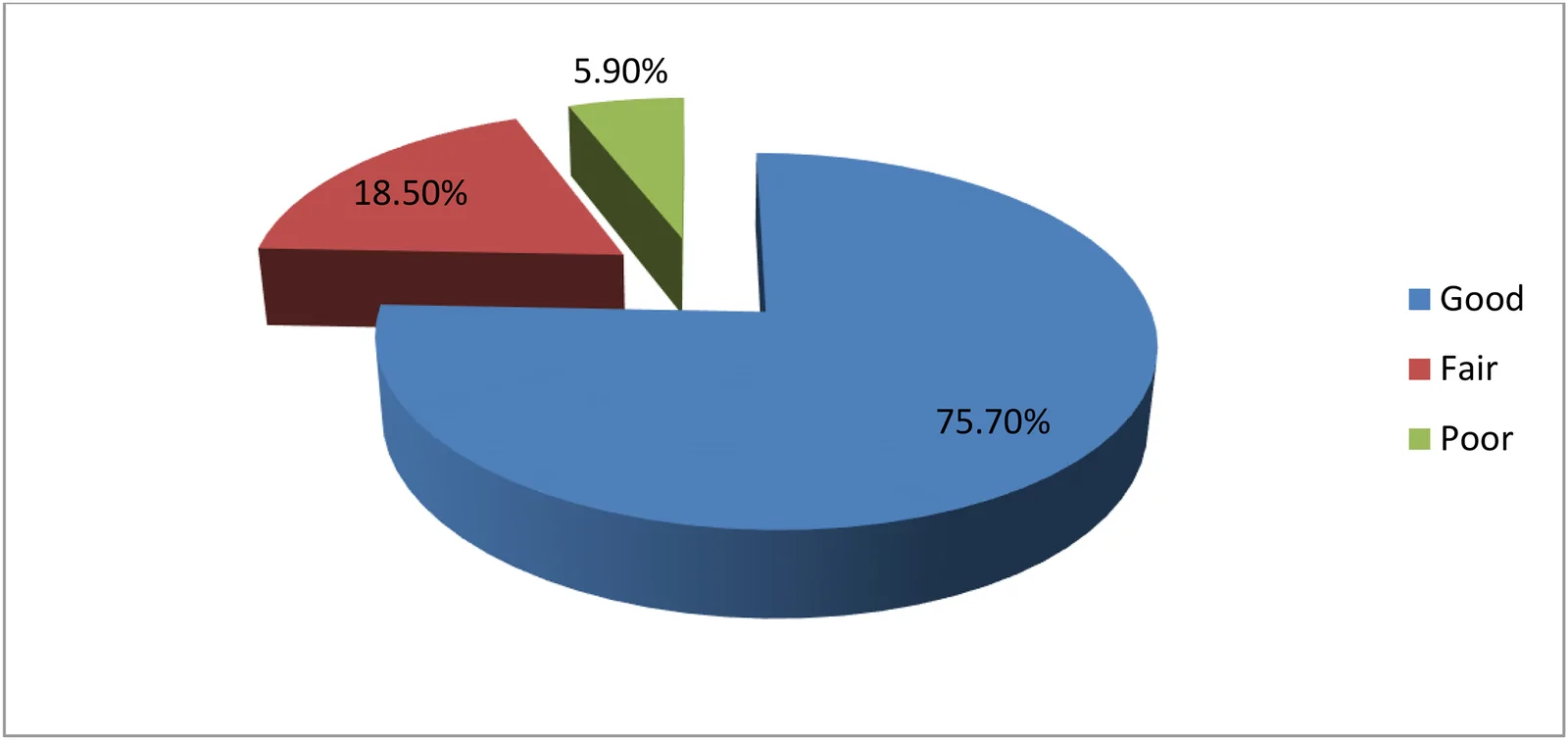
PMTCT adherence among HIV-exposed infants during follow-up in Tigray, Ethiopia, 2020.

### Bivariate and multivariable analysis of MTCT of HIV

The relationship between the independent variables and the incidence of MTCT of HIV was analyzed by using the logistic regression model. In the bivariate logistic regression analysis, the independent variables mother’s place of residence, occupation of mother, mothers enrolled to PMTCT during labor and delivery or after, mothers not disclosed their HIV status to others, a mother with the detectable viral load during pregnancy, maternal nutritional status during pregnancy, age of infant >6 weeks during enrolment to PMTCT, poor adherence of the infant for PMTCT drugs, father’s not enrolled to ART, advanced WHO clinical stage of the mother at baseline were all associated with mother to child transmission of HIV.

In multivariable logistic regression analysis, poor adherence of infant for PMTCT, father’s not enrolled to ART, > 6 weeks age of the infant at enrolment to PMTCT, mother enrollment

to PMTCT during labor and delivery or after and mothers on advanced WHO clinical stage at baseline were found to be the most important significant predictors of mother-to-child transmission of HIV.

The odds of having HIV positive results among HIV exposed infants with poor adherence for PMTCT drugs was 5.6 compared with good adherence (AOR: 5.6; 95% CI: 1.020–30.249). The odds of having HIV positive test results among HIV exposed infants whose HIV positive father was not enrolled in ART was 4.4 compared to those their fathers enrolled in ART (AOR: 4.4; 95% CI: 1.2–16.7). The odds of having HIV positive test result among HEIs enrolled to PMTCT at the age of >6 weeks was also 4.4 compared with these enrolled early (AOR: 4.4; 95% CI: 1.2–17.0). The odds of having HIV positive infant among mothers enrolled to PMTCT during labor and delivery or after was also 7.9 compared with enrolled to ART before pregnancy (AOR: 7.9; 95% CI: 1.4–44.7) and the odds of having HIV positive infant among mothers on advanced WHO clinical stage at the baseline was 4.2 compared with the mothers on mild stage (AOR: 4.2; 95% CI: 1.0–17.8) (Table 5).

**Table 5:** Multivariable analysis of predictors of MTCT of HIV, in public general hospitals of Tigray, Ethiopia May 2020.

Variables	Category	HIV test result of HEI		COR (95% CI)	AOR(95% CI)	P value
		Positive (%)	Negative (%)			
Residence	Rural	6 (5.9)	96 (94.1)	1.8(0.7-5.2)	1.9 (.45 −7.9)	.380
	Urban	11(3.2)	331(96.8)		1	
Occupation	Unemployed	11 (5.2)	199(94.8)	2.1(0.7 −5.7)	.7 (.17 - 2.9)	0.655
	Employed	6(2.6)	228 (97.4)		1	
Time of Enrollment to PMTCT	During L&D or after	6 (14.3)	36(85.7)	7.3(2.2 −23.9)	7.9(1.4-44.7)	0.018*
	During pregnancy	5(3.8)	126 (96.2)	1.7 (0.52 - 5.8)	4(0.8 - 20.1)	.084
	Before pregnancy on HAART	6(2.2)	264(97.8)		1	
Disclosure status	No	9(8.2)	101 (91.8)	3.6 (1.36- 9.6)	3.2(0.9- 12.2)	0.075
	Yes	8(2.4)	326 (97.6)		1	
Last viral load during pregnancy	Detectable	7 (8.0)	81 (92)	2.99(1.1 - 8.)	1.07(.2 - 4.9)	0.928
	Undetectable	10 (2.8)	346 (97.2)		1	
Nutritional status during pregnancy	Malnourished	8(8.8)	83 (91.2)	3.68 (1.3–9.8)	1.6 (.4 - 6.5)	0.490
	Not malnourished	9 (2.5)	344 (97.5)		1	
Age of infant in weeks at enrollment	>6weeks	10 (12.2)	72 (87.8)	7.04(2.6- 19.1)	4.5 (1.2- 17)	0.029*
	<=6weeks	7 (1.9)	355 (98.1)		1	
Adherence of HEI for PMTCT	Poor	5 (19.2)	21 (80.8)	11(3.2 - 38.2)	5.6 (1.- 30.2)	0.047*
	Fair	5 (6.1)	77(93.9)	3 (0.94 - 9.87)	3.9 (.8 - 17.6)	0.078
	Good	7 (2.1)	329(97.9)		1	
Father’s enrollment to ART	No	8 (16.7)	40 (83.3)	6.6 (2.3- 18.6)	4.5(1.2 - 16.7)	0.026*
	Yes	8 (2.9)	264 (97.1)		1	
WHO clinical stage of mother at baseline	Advanced stage	9(10.2)	79 (89.8)	4.95(1.8- 13.2)	4.2 (1. - 17.9)	0.047*
	Mild stage	8 (2.2)	348 (97.8)		1	

## Discussion

This hospital-based retrospective study assessed the incidence and predictors of MTCT of HIV among HIV exposed infants in public General Hospitals of Tigray Regional State.

The cumulative incidence of MTCT of HIV in this study was 3.8% (95% CI: 2.3–5.8), indicating that HIV transmission from mother to child remains a public health concern in the study area. This finding is comparable with studies conducted in Kenya (5%) and Bahir Dar, Ethiopia (5.8%) [13,27].

The result of this study was lower than the finding of retrospective studies conducted in Cameron (7.1%), Kenya (9.3%), Democratic Republic of Congo (16.3%), Brazil (8.2%), Uganda(6.5%), India(7.8%), in Ethiopia (Amhara Region South Gondar Zone (10.1%), Dire Dawa(15.7%), Gondar University Referral Hospital (10%), Saint Merry Luke Referral Hospital (9.6%), Asella Hospital (7.7%), East and West Gojam Zone of Amhara Region (5.9%,)) [14,17,18,21,28–35]. This difference might be due to the time difference between the studies, in which this study was conducted at a time of high coverage of ART for pregnant and lactating women, ARV prophylaxis for HEI, ANC follow up, and institutional delivery than the previous studies. In this study, 99.5% of the mothers were enrolled to ART but in the other studies conducted before only 24.4% in Southwest Ethiopia Saint Merry Luke Referral Hospital to the maximum of 81.2% of the mothers were enrolled to ART in Gondar University Referral Hospital northwest Ethiopia [14,18,21,33].The other reason might be also ARV prophylaxis at birth in this study were high (94.1%) compared to the other studies conducted before which was only 31.7% to the maximum of 85.1% in Gondar University Referral Hospital northwest Ethiopia [14,18,21,29–31,35].

In this study, 97.3% of the mothers were delivered at health institution compared with the previous studies conducted which were 58.7% in southwest Ethiopia to a maximum of 90.8% in Assella Hospital [14,18,21,30,32,35]. This may help to enroll the HEIs to PMTCT service at birth or as early as possible and can have a significant positive effect on reduction of MTCT of HIV. In this study none of the HIV-exposed infants received mixed feeding, but in the other studies before significant number of HEI(7.9% −20.7%) were on mixed feeding practice before six months of age [14,18,21,29,35]. Mixed feeding practice before six months of age may cause diarrheal diseases and infection of gastro intestinal system and can increases the risk of MTCT of HIV.

Because this study was conducted at the time of all pregnant and lactating mothers with HIV enrolled to ART regardless of their WHO clinical staging and CD4 count and the data of most of the studies conducted before were before the implementation of option B+, [14,18,21,30,33,35] this may had significant effect on reduction of MTCT of HIV. Additionally most of the studies conducted before were in single health institutions [14,18,21,35].

This result was higher compared with the retrospective cohort study in Nigeria, prospective study conducted in Nigeria in 2015, cross-sectional study conducted in Tigray Region in 2016 that the rate of MTCT of HIV were 0.7%, 1%, and 2.1% respectively [12,36,37]. This difference might be due to the sample size of the retrospective study conducted in Nigeria and a cross-sectional study conducted in Tigray was small compared to this study. Additionally, the data of the study conducted in Tigray was taken from a short period from September 01 to December 30, 2016 [12].The other reason might be all the study participants in Nigeria were on ANC, delivered at health institution, and all the HEIs were enrolled to PMTCT before 72 hours [36].

In this study, HEI enrolled in PMTCT at the age of >6 weeks were 4.5 times at risk to be HIV positive than their counterparts (95% CI; 1.2–17.0 P-value.029). This result was supported by the study conducted in Gondar, Ethiopia in 2013 and Uganda in 2018 [18,32]. This might be due to the reason that 10–15% and 5–15% of MTCT of HIV is during labor and delivery and breastfeeding respectively, early initiation of ARV prophylaxis for all HIV exposed infants can significantly reduce MTCT of HIV that occurs after delivery [5,38].

HEI whose mothers enrolled in PMTCT follow up during labor and delivery or after were 7.9 times more likely to be HIV positive than those their mothers were on HAART before pregnancy (95% CI, 1.4–44.7, P-value.018). This result was supported by the study conducted in East and West Gojam Zones, retrospective follow up studies conducted in Gondar Ethiopia, and Cameron [12,17,18,28]. This is due to the reason that early detection and initiation of ARV drugs for HIV positive mothers can significantly reduce the chance of the child from becoming infected with HIV [5]. Additionally, HIV positive mothers on chronic ART have a low viral load, less opportunistic infections, and they have better knowledge on prevention mechanisms so that there is less probability of transmitting to the child through pregnancy, childbirth, and breastfeeding.

HEI who had poor adherence for PMTCT were also 5.6 times more likely to be HIV positive than those with good adherence (95% CI: 1.0–30.2, P-value 0.047). This can be supported with the advantage of ARV prophylaxis for HIV exposed infants [1].

HEI born from mothers with advanced WHO clinical stage at baseline were also 4.2 times more likely to be HIV positive than those born from mothers on mild stages at baseline (95% CI 1.0–17.8, P-value.047). This finding was supported with the study conducted in South West Ethiopia in 2014 [21]. It is also supported by PMTCT guide lines that suggests mothers on advanced WHO clinical stage are at risk to have HIV positive infants [5]. This may be due to the reason that HIV positive mothers with advanced WHO clinical stage, may have a high viral load, and different opportunistic infections; this may increase the risk of MTCT of HIV.

HEI whose fathers were HIV positive and not enrolled in ART was also 4.5 times at risk to be HIV positive than these HIV exposed infants whose fathers were enrolled in ART service (95% CI 1.2–16.7 P-value 0.026). The reason for this result might be, the mothers whose husbands were not enrolled in ART may not have an open discussion and they may not get support from their husbands.

### Strength and limitations

#### Strength.

The strengths of this study were, this study was conducted in the relatively large study area and sample size compared with the studies conducted in Ethiopia before, the other strength of this study was also variables like enrollment of HIV positive father to ART, viral load of the mother, and duration of breastfeeding that were not addressed by most other studies were addressed.

#### Limitation.

This study had some limitations. Because this study was retrospective study, the variables duration of the rapture of the membrane, duration of labor, and income of the family were not addressed due to most of the charts were not documented. This study did not differentiate when most the HIV exposed infants were infected with HIV, i.e., during pregnancy, labor and delivery or breastfeeding period. HIV infected mothers who are not tested for HIV and those HIV exposed infants not registered to PMTCT follow up might be also missed.

### Conclusion

The cumulative MTCT rate of 3.8% demonstrates substantial progress toward elimination targets in breastfeeding populations, although continued improvement is needed to achieve the Ethiopian national target of zero new pediatric HIV infections. **Poor infant adherence** for PMTCT drugs, HIV positive father not enrolled to ART, infant enrollment into PMTCT after 6 weeks of age, mothers enrolled to PMTCT during labor and delivery or after and mothers on advanced WHO clinical stage at baseline were significantly associated with MTCT of HIV.

## Abbreviations

ANC: Antenatal Care
AOR: Adjusted Odds Ratio
ART: Antiretroviral Therapy
ARV: Antiretroviral
COR: Crude Odds Ratio
CPT: Cotrimoxazole Preventive Therapy
DNA: Deoxyribonucleic Acid
EBF: Exclusive Breast Feeding
EDHS: Ethiopian Demographic and Health Survey
ERF: Exclusive Replacement Feeding
FMOH: Federal Ministry of Health
HAART: Highly Active Antiretroviral Therapy
HIV: Human Immunodeficiency Virus
HEI: HIV Exposed
MTCT: Mother-To-Child Transmission of HIV
NVP: Nevirapine
PCR: Polymerase Chain Reaction
PMTCT: Prevention of Mother-To-Child Transmission
WHO: World Health Organization
