# Supplementary material for: Incidence and predictors of mother-to-child transmission of HIV among HIV-exposed infants in public General hospitals of Tigray, Ethiopia, 2020: Retrospective cohort study
Source: PLoS One. 2026 Oct 22;21(10):e0358878. doi: 10.1371/journal.pone.0358878 (PMC13690227; doi:10.1371/journal.pone.0358878)
Supplement: Supplementary file 1 [file pone.0358878.s002.docx]

# **Response to comments of editors and Reviewers**

## 1. ****Journal Requirement****

### Please ensure that your manuscript meets PLOS ONE's style requirements, including those for file naming.

**Response:** We thank the editor for this reminder. We have carefully reviewed the manuscript and revised it to comply with the PLOS ONE formatting and style requirements. We have also ensured that all submitted files, including the revised manuscript, the tracked-changes version, and the Response to Reviewers document, follow the journal's file naming and formatting guidelines

. 2. Editor's Comment

We note that your Data Availability Statement is currently as follows: "All relevant data are within the manuscript and in Supporting Information files." Please confirm at this time whether or not your submission contains all raw data required to replicate the results of your study. Authors must share the "minimal data set" for their submission... If your submission does not contain these data, please either upload them as Supporting Information files or deposit them in a stable, public repository. If there are ethical or legal restrictions on sharing a de-identified data set, please explain them in detail.

**Response:** Thank you for bringing this important issue to our attention. We sincerely apologize for inadvertently submitting the original SPSS data file containing potentially identifying information in the Supporting Information.

We acknowledge that the protection of participant privacy is essential when sharing human participant data. The original SPSS file has therefore been withdrawn and will not be resubmitted in its original form.

We have reviewed the dataset and prepared a de-identified minimal dataset containing only the variables necessary to reproduce the analyses and findings reported in the manuscript. Direct identifiers and potentially identifying information, including names, medical record numbers, specific dates, contact information, and other identifiers, are removed. We have also reviewed combinations of indirect identifiers to minimize the possibility of participant re-identification.

**Variables included in the de-identified minimal dataset and their contribution to the reported analyses**

| **No.** | **Variable** | **Categories/measurement used in analysis** | **Role in analysis** | **Table/analysis** |
| --- | --- | --- | --- | --- |
| 1 | HIV test result of HEI | Positive / Negative | **Outcome variable** | Tables 2–5; incidence and logistic regression |
| 2 | Maternal age | 15–24; 25–34; ≥35 years | Independent variable | Table 2 |
| 3 | Residence | Urban / Rural | Independent variable | Tables 2 & 5 |
| 4 | Maternal occupation | Employed / Unemployed | Independent variable | Tables 2 & 5 |
| 5 | Religion | Orthodox / Muslim / Other | Descriptive variable | Table 2 |
| 6 | Marital status | Married / Single / Divorced/Widowed | Descriptive variable | Table 2 |
| 7 | Educational status | Not educated / Primary / Secondary and above | Descriptive variable | Table 2 |
| 8 | WHO clinical stage at baseline | Mild (I–II) / Advanced (III–IV) | Independent variable | Tables 3 & 5 |
| 9 | CD4 count at baseline | ≥500; 351–499; 200–350; <200 cells/mm³ | Independent variable | Table 3 |
| 10 | Last CD4 count during pregnancy | ≥500; 351–499; 200–350; <200 cells/mm³ | Independent variable | Table 3 |
| 11 | Functional status at baseline | Working / Ambulatory / Bedridden | Independent variable | Table 3 |
| 12 | Time of maternal HIV diagnosis | Before pregnancy / During pregnancy or after | Independent variable | Table 3 |
| 13 | Maternal PMTCT enrollment | Before pregnancy on HAART / During pregnancy / During labor and delivery or after* | Independent variable | Tables 3 & 5 |
| 14 | Maternal ART adherence | Good / Fair / Poor | Independent variable | Table 3 |
| 15 | Opportunistic infection at baseline | Yes / No | Independent variable | Table 3 |
| 16 | HIV disclosure status | Yes / No | Independent variable | Tables 3 & 5 |
| 17 | Last maternal viral load during pregnancy | Undetectable / Detectable | Independent variable | Tables 3 & 5 |
| 18 | Maternal nutritional status during pregnancy | Not malnourished / Malnourished | Independent variable | Tables 3 & 5 |
| 19 | ANC follow-up | Yes / No | Independent variable | Table 4 |
| 20 | Number of ANC visits | ≥4; 2–3; 1 | Independent variable | Table 4 |
| 21 | Postnatal follow-up | Yes / No | Independent variable | Table 4 |
| 22 | Gravidity | I / II / III and above | Independent variable | Table 4 |
| 23 | Parity/number of children | 1 / 2 / ≥3 | Independent variable | Table 4 |
| 24 | Place of delivery | Health institution / Home | Independent variable | Table 4 |
| 25 | Mode of delivery | SVD / CS | Independent variable | Table 4 |
| 26 | Infant sex | Male / Female | Independent variable | Table 4 |
| 27 | Infant birth weight | ≥2.5 kg / <2.5 kg | Independent variable | Table 4 |
| 28 | Infant growth pattern | Normal / Growth failure | Independent variable | Table 4 |
| 29 | Feeding practice before 6 months | EBF / ERF / Mixed feeding | Independent variable | Table 4 |
| 30 | Feeding practice after 6 months | Breastfeeding + complementary feeding / Stop breastfeeding | Independent variable | Table 4 |

**3.** Your ethics statement should only appear in the Methods section of your manuscript. If your ethics statement is written in any section besides the Methods, please move it to the Methods section and delete it from any other section. Please ensure that your ethics statement is included in your manuscript, as the ethics statement entered into the online submission form will not be published alongside your manuscript.

**Response:** We thank the editor for highlighting this formatting requirement. In accordance with *PLOS ONE* guidelines, we have reviewed the entire manuscript, ensured that the complete ethics statement appears exclusively within the Methods section under the dedicated subsection, and deleted duplicate declarations from any other sections.

**4.** If the reviewer comments include a recommendation to cite specific previously published works, please review and evaluate these publications to determine whether they are relevant and should be cited. There is no requirement to cite these works unless the editor has indicated otherwise

### Response: We sincerely thank the reviewer for pointing out these important, recently published studies from 2024 and 2025.

We recognize that these papers were published after our data collection and baseline window closed around 2020. However, in accordance with standard academic peer-review practices, we have carefully evaluated these recent publications and found them to be exceptionally relevant. Incorporating these newer systematic reviews and regional studies allows us to effectively bridge our historical pre-conflict baseline data with contemporary evidence, demonstrating how our findings compare with broader post-2020 trends across Ethiopia

## Response to Reviewers

## Reviewer 1

### Comment 1

The title could be Incidence and predictors of mother-to-child transmission of HIV among HIV-exposed infants in Tigray, Ethiopia: A retrospective cohort study (consider either public or general hospital).

### Response: Thank you for this helpful suggestion. We agree that the original title could be made clearer and more concise. In response to your comment, we have revised the title by removing the phrase "attending in" and retaining "public general hospitals" to accurately reflect the study setting, as the study was conducted exclusively in public general hospitals in Tigray. The revised title now reads:

### Comment 2

In the abstract, the last paragraph of the background should be rephrased for clarity.

Response**:**Thank you for this helpful suggestion. We agree that the final part of the abstract background could be expressed more clearly. Accordingly, we have revised this section to improve its clarity, readability, and logical flow by better linking the existing research gap with the objective of the study

### Comment 3

Correct grammar in lines 79 and 80.

### Response: Thank you for identifying this grammatical issue. We have carefully revised the text in lines 79–80 to improve grammar and readability. The sentence has been corrected accordingly in the revised manuscript

### Comment 4

In lines 101 and 102, it may be necessary to cite some of the other studies.

## **Response**: Thank you for this valuable suggestion. We agree that the statement in lines 101–102 would be strengthened by citing additional relevant studies. Accordingly, we have revised this section by incorporating appropriate references from previous studies to provide broader evidence and better support the statement. The revised manuscript now includes these additional citations in lines 101–102.

### Comment 5

Rephrase line 138

### Response: Thank you for this helpful suggestion. We have revised the paragraph to improve its clarity, readability, and grammatical accuracy while preserving its original meaning.

### Comment 6

In the section of independent variables, father HIV status and Enrolment to ART an infant factor?

Response: Thank you for this valuable observation. We agree with the reviewer that **father HIV status** and **father enrollment in antiretroviral therapy (ART)** are paternal characteristics rather than infant factors. Their placement under the "Infant factors" category was an oversight during manuscript preparation. We have revised the Methods section by creating a separate category entitled **Paternal factors**, under which **father HIV status** and **father enrollment in ART** are now appropriately listed. This correction improves the clarity and conceptual organization of the independent variables without affecting the study design, analysis, or findings.

Comment 7:
It might be necessary to cite some of the resources from where the data extraction form was adapted from.

Response: Thank you for this valuable comment. We agree that the sources used to develop and adapt the data extraction form should be clearly cited. Accordingly, the relevant references have been added to the Methods section and incorporated into the reference list.

### Comment 8

What was the detailed information on the records and management of the infants obtained from key informants (health care providers) working at the PMTCT unit used for as indicated in lines 243 and 244?

Response**:** Thank you for this important comment. The healthcare providers working in the PMTCT units were **not** interviewed to obtain additional study data. Their role was limited to assisting the research team in locating medical records, clarifying the organization and documentation of the PMTCT registers, and resolving inconsistencies or missing information identified during data extraction from the medical records. No information obtained directly from the healthcare providers was included in the study dataset or statistical analysis.

### Comment 9

In line 256, for clarity it may be necessary to indicate the modifications done on the data extraction form after pre testing

**Response:** Thank you for this valuable suggestion. We have revised the manuscript to clearly describe the modifications made to the data extraction form following the pretest.

### Comment 10

From line 256, I am not sure how pre testing was used to later check for completeness and accuracy before both the data collectors and supervisors go to the field

**Response:** Thank you for this insightful comment. We agree that our original wording was unclear and may have implied that the pretest itself was used to assess completeness and accuracy. Our intention was to describe two separate quality control procedures. First, the pretest was conducted to identify ambiguities, missing items, and other practical issues in the data extraction form, and the tool was subsequently revised accordingly. Second, the revised version of the data extraction form was independently reviewed by the investigators, supervisors, and data collectors to ensure its completeness and accuracy before the commencement of data collection. We have revised the manuscript to clarify this sequence.

### Comment 11

Rephrase line 274 and 275 for clarity

**Response:** Thank you for this helpful suggestion. We have revised the operational definition of mother-to-child transmission (MTCT) of HIV to improve its clarity and readability

### Comment 12

 Check spelling of last on table 3

**Response:** Thank you for pointing this out. We carefully reviewed Table 3 and corrected the spelling and formatting errors.

Comment 13

Be consistent with the use of either British or American English

**Response:** Thank you for this valuable suggestion. We have carefully reviewed the entire manuscript and revised the language to ensure consistent use of British English throughout. Spelling, terminology, and grammatical conventions have been standardized accordingly.

## Reviewer 2

### Comment 1

Although the study sample includes HEIs who registered in PMTCT (the majority at the age of 6 weeks), there is no mention of HEI loss to follow-up, proportion of HEIs who received recommended infant virologic testing, or HEIs who received the final test after breastfeeding cessation. Given the high rates of LTFU for HEIs, the very low coverage of EID in Ethiopia for the 6 week test (and even lower coverage for the 9 month virologic test), the denominator of HEIs used for the MTCT rate is inherently inaccurate and unreliable.

**Response:**We sincerely thank the reviewer for this important observation. We agree that loss to follow-up (LTFU) and completion of the recommended early infant diagnosis (EID) testing schedule are important considerations when interpreting MTCT estimates.

Our study was conducted as a retrospective cohort review using routinely collected PMTCT records from six public General Hospitals in Tigray. The study included only HIV-exposed infants with a documented final HIV outcome, defined as either a DNA-PCR result before 18 months of age or a final rapid antibody test performed at least six weeks after cessation of breastfeeding. Infants without documented final HIV test results were excluded according to our predefined eligibility criteria. Consequently, infants who were lost to follow-up before completion of HIV testing were not included in the analytical cohort.

To improve clarity, we have revised the Methods section to explicitly describe the eligibility criteria and the derivation of the final analytical sample. We have also clarified in the Results that among the 485 sampled mother–infant pairs, 444 (91.5%) had complete HIV outcome data and were included in the analysis, while 41 (8.5%) records were excluded because the final HIV test result was not documented. In addition, we have expanded the Limitations section to acknowledge that excluding infants without documented final HIV outcomes may have introduced selection bias and could have resulted in underestimation or overestimation of the true MTCT rate because infants lost to follow-up were not represented.

Because the available retrospective records did not consistently document the timing and completeness of all recommended virologic tests, we were unable to accurately report coverage of each recommended test. We have acknowledged this limitation explicitly in the revised manuscript.

### Comment 2

In Table 3, there is no delineation of missing data for CD4 and viral load testing. This is not believable for any clinical setting (especially one that is a retrospective review of routine program data). Coverage of viral load testing in pregnant women in recent data was under 70% and casts doubt on the credibility of this and all other data tables presented.

**Response:**We thank the reviewer for highlighting this important issue. We agree that retrospective programmatic data commonly contain missing laboratory information.

Following the reviewer's suggestion, we have re-examined the original medical records and data extraction forms. In the revised manuscript, we have reported the extent of missing data for laboratory variables, including maternal CD4 count and viral load measurements, wherever applicable. Table 3 has been revised to clearly distinguish documented laboratory results from missing observations. We have also clarified in the Methods section how missing values were handled during data analysis.

Where laboratory information was unavailable in the medical records, those observations were treated as missing and were not imputed. The number of observations included in each analysis therefore reflects the availability of documented data.

**Comment 3**

Missing data (clinical, laboratory, and engagement in care/retention) is critical for these types of programmatic analyses and is ignored throughout.

**Response**

We appreciate the reviewer's insightful comment. We agree that reporting missing data improves transparency in retrospective analyses.

Accordingly, we have revised the manuscript to describe the completeness of routinely collected clinical and laboratory variables and have added a statement in the Methods explaining our approach to missing data. Variables with unavailable documentation were treated as missing and were not imputed.

### Comment 4

There are numerous typographical and word errors throughout.

**Response:**We sincerely thank the reviewer for this observation. The entire manuscript has been carefully edited for English language, grammar, spelling, punctuation, terminology, and sentence structure. We also corrected typographical errors, improved scientific wording, and standardized terminology throughout the manuscript to improve clarity and readability.

**Comment 5**

Several pieces of epidemiologic data and references within the introduction are severely outdated (e.g., 2016–2018) despite availability of more recent estimates.

**Response:**We thank the reviewer for this valuable suggestion. We agree that the Introduction should reflect the most recent epidemiological evidence.

Accordingly, we have substantially revised the Introduction by updating global, regional, and Ethiopian HIV epidemiological data using the most recent WHO, UNAIDS, and national reports available. Older references have been retained only when they provide historical context or support statements that remain relevant. The revised Introduction now better reflects the current epidemiology of HIV and progress toward elimination of mother-to-child transmission.

## Reviewer 3

We sincerely thank the reviewer for the thoughtful, constructive, and encouraging comments. We greatly appreciate the recognition of the importance of our study, the rigor of the methodology, and the overall quality of the manuscript. We have carefully considered all suggestions and revised the manuscript accordingly.

### Comment 1

### The study is by and large well-written, though some slight editing is needed especially in the abstract and introduction, and well-referenced.

### Response**:**

### We sincerely thank the reviewer for the positive evaluation of our manuscript and for this helpful suggestion. In response, we have carefully revised the entire manuscript, with particular attention to the Abstract and Introduction. The language has been improved for clarity, grammar, and readability, and several sentences have been restructured to improve the overall flow. We have also updated and expanded the reference list where appropriate.

### Comment 2

Unfortunately, the study is rather out-of-date. It would appear that the study was started before the war in Tigray started, and could not be concluded until well after it ended.

### Response**:** We thank the reviewer for this thoughtful observation. We agree that there is a substantial interval between data collection and submission of the manuscript.

The study was conducted using retrospective data collected in early 2020, immediately before the armed conflict in the Tigray Region. Soon after data collection, the conflict resulted in prolonged disruption of health services, displacement of researchers, destruction of institutional infrastructure, and an almost complete interruption of communication and internet services. These circumstances substantially delayed data analysis, manuscript preparation, revision, and submission.

To improve transparency, we have added an explanation in the Discussion describing the timing of the study and the exceptional circumstances that contributed to the delay in publication. We believe that, despite the time interval, these data remain valuable because they provide an important pre-conflict baseline for PMTCT program performance in Tigray. Such baseline information is essential for evaluating the subsequent impact of the conflict on maternal and child HIV services and for informing future recovery efforts.

### Comment 3

Many of the references are from 2015–2018; the data for PMTCT and HIV globally could easily have been updated to 2024 from UNICEF, UNAIDS or WHO.

### Response**:** We appreciate this valuable recommendation and fully agree. Accordingly, we have comprehensively updated the introduction and discussion using the most recent epidemiological reports and scientific literature available from WHO, UNAIDS, UNICEF, and recent peer-reviewed publications.

### Comment 4

Unless the authors specifically state that this is a historical snapshot from 2020, the article has little relevance to today.

### Response**:** We thank the reviewer for this excellent suggestion. We agree that the context of the study should be made clearer.

Accordingly, we have revised the Introduction and Discussion to explicitly state that the study represents a **pre-conflict (2020) assessment** of PMTCT services in Tigray. We also explain that these findings provide an important historical baseline against which post-conflict PMTCT service recovery and future program performance can be evaluated. We believe this clarification substantially strengthens the relevance and interpretation of our findings.

### Comment 5

I will leave it to the authors to compare their 2020 findings to more recent findings and to explain the time-lag.

### Response**:** We sincerely thank the reviewer for this insightful recommendation. In response, we have expanded the Discussion by comparing our findings with more recent studies conducted in Ethiopia and other sub-Saharan African countries, including recent systematic reviews and studies published after 2020. We have also included a brief explanation regarding the unavoidable delay between data collection and publication due to the armed conflict in Tigray. These additions provide readers with an updated perspective on the study findings while emphasizing the continuing importance of the 2020 baseline data.

## Reviewer 4

### Comment 1

The study is described as a retrospective cohort, yet the analysis relies solely on binary logistic regression with no time-to-event consideration. This raises concerns whether incidence is appropriately measured.

**Response:** Thank you for this important observation.

We agree that survival analysis is the preferred method when exact event times and censoring information are available. However, our study was based on retrospective review of routine PMTCT records. The exact timing of HIV acquisition was unavailable because infants were tested at different scheduled intervals (DNA-PCR and final antibody testing), and the precise date of infection could not be determined.

### Comment 2

Confounding control is limited.

**Response:** Thank you.

Potential confounders including maternal viral load, CD4 count, maternal nutritional status, disclosure status, WHO clinical stage, ART adherence, ANC follow-up and infant-related factors were evaluated during bivariable analysis.

Variables with p<0.25 were entered into the multivariable logistic regression model.

Maternal viral load and several other variables were included in the initial multivariable model but were not statistically significant after adjustment and therefore were not retained in the final model. We have clarified this process in the revised Methods and Results sections.

### Comment 3

Wide confidence intervals suggest model instability.

**Response:** We appreciate this observation.

Only 17 MTCT events occurred among the 444 HIV-exposed infants, resulting in relatively few outcome events.

The small number of events inevitably reduced the precision of some estimates and resulted in wider confidence intervals.

### Comment 4

Inclusion criteria may introduce selection bias.

**Response:** We agree.

Only infants with documented HIV outcomes were included because confirmation of HIV status was required for the primary outcome assessment.

This may have excluded infants lost to follow-up or transferred out, potentially introducing selection bias.

## Methodological Clarity

**Comment:** Why were only 40% of hospitals selected?

**Response:** Thank you.

Fourteen public general hospitals were providing PMTCT services during the study period.

Due to resource constraints, time, and logistics, six hospitals (approximately 40%) were selected using simple random sampling (lottery method).

Following hospital selection, proportional allocation and simple random sampling of patient records were performed.

**Comment**

Exposure period and follow-up are unclear.

**Response:** Thank you.

The follow-up period extended from enrollment in PMTCT until the infant's final HIV status was confirmed by DNA-PCR or antibody testing after cessation of breastfeeding.

Because exact dates of HIV acquisition were unavailable, person-time and survival analysis could not be performed.

**Comment**

Missing data handling was not described.

**Response:** Thank you.

Before analysis, all extracted data were checked for completeness and consistency.

Records lacking confirmed HIV outcomes were excluded according to the predefined eligibility criteria.

Variables with missing values were evaluated during data cleaning.

## Interpretation

**Comment**

The conclusion that MTCT is "still high" is overstated.

**Response:** Thank you, the conclusion has been revised to better reflect the findings.

### Comment

Interpretation regarding paternal ART is speculative.

**Response:** Thank you; we have revised the discussion to clearly indicate that the proposed mechanisms are hypotheses rather than direct evidence from this study.

We now state that paternal ART enrollment may reflect greater family engagement, treatment adherence, and support for maternal PMTCT, but further prospective studies are required to establish causal pathways.

- Language and Scientific Writing Comments
  • The manuscript requires substantial language editing:
  o Frequent grammatical errors
  o Inconsistent tense usage
  • Some statements lack precision (e.g., “this study was identified”).

### Response: Thank you.The entire manuscript has undergone comprehensive English language editing to improve grammar, scientific style, consistency, and readability.
