# Supplementary material for: Incidence and predictors of mother-to-child transmission of HIV among HIV-exposed infants in public General hospitals of Tigray, Ethiopia, 2020: Retrospective cohort study
Source: PLoS One. 2026 Oct 22;21(10):e0358878. doi: 10.1371/journal.pone.0358878 (PMC13690227; doi:10.1371/journal.pone.0358878)
Supplement: Peer review history

# Peer review: Decision Letter 0

**DOI:** 10.1371/journal.pone.0358878.r001

**Authors:** David Ikwuka

22 Jun 2026

Dear Dr. Gerezgiher,

Thank you for submitting your manuscript to PLOS ONE. After careful consideration, we feel that it has merit but does not fully meet PLOS ONE’s publication criteria as it currently stands. Therefore, we invite you to submit a revised version of the manuscript that addresses the points raised during the review process.

Please submit your revised manuscript by Aug 06 2026 11:59PM. If you will need more time than this to complete your revisions, please reply to this message or contact the journal office at plosone@plos.org. When you're ready to submit your revision, log on to https://www.editorialmanager.com/pone/ and select the 'Submissions Needing Revision' folder to locate your manuscript file.

- A letter that responds to each point raised by the academic editor and reviewer(s). You should upload this letter as a separate file labeled 'Response to Reviewers'.
- A marked-up copy of your manuscript that highlights changes made to the original version. You should upload this as a separate file labeled 'Revised Manuscript with Track Changes'.
- An unmarked version of your revised paper without tracked changes. You should upload this as a separate file labeled 'Manuscript'.

If you would like to make changes to your financial disclosure, please include your updated statement in your cover letter. Guidelines for resubmitting your figure files are available below the reviewer comments at the end of this letter.

If applicable, we recommend that you deposit your laboratory protocols in protocols.io to enhance the reproducibility of your results. Protocols.io assigns your protocol its own identifier (DOI) so that it can be cited independently in the future. For instructions see: https://journals.plos.org/plosone/s/submission-guidelines#loc-laboratory-protocols. Additionally, PLOS ONE offers an option for publishing peer-reviewed Lab Protocol articles, which describe protocols hosted on protocols.io. Read more information on sharing protocols at https://plos.org/protocols?utm_medium=editorial-email&utm_source=authorletters&utm_campaign=protocols.

As the corresponding author, your ORCID iD is verified in the submission system and will appear in the published article. PLOS supports the use of ORCID, and we encourage all coauthors to register for an ORCID iD and use it as well. Please encourage your coauthors to verify their ORCID iD within the submission system before final acceptance, as unverified ORCID iDs will not appear in the published article. *Only*  the individual author can complete the verification step; PLOS staff *cannot*  verify ORCID iDs on behalf of authors.

We look forward to receiving your revised manuscript.

Kind regards,

David Chibuike Ikwuka, Ph.D.

Academic Editor

PLOS One

Journal Requirements:

When submitting your revision, we need you to address these additional requirements.

1.Please ensure that your manuscript meets PLOS ONE's style requirements, including those for file naming. The PLOS ONE style templates can be found at

https://journals.plos.org/plosone/s/file?id=wjVg/PLOSOne_formatting_sample_main_body.pdf and

https://journals.plos.org/plosone/s/file?id=ba62/PLOSOne_formatting_sample_title_authors_affiliations.pdf

2. We note that your Data Availability Statement is currently as follows: All relevant data are within the manuscript and in Supporting Information files.

Please confirm at this time whether or not your submission contains all raw data required to replicate the results of your study. Authors must share the “minimal data set” for their submission. PLOS defines the minimal data set to consist of the data required to replicate all study findings reported in the article, as well as related metadata and methods (https://journals.plos.org/plosone/s/data-availability#loc-minimal-data-set-definition).

For example, authors should submit the following data:

- The values behind the means, standard deviations and other measures reported;

- The values used to build graphs;

- The points extracted from images for analysis.

Authors do not need to submit their entire data set if only a portion of the data was used in the reported study.

If your submission does not contain these data, please either upload them as Supporting Information files or deposit them to a stable, public repository and provide us with the relevant URLs, DOIs, or accession numbers. For a list of recommended repositories, please see https://journals.plos.org/plosone/s/recommended-repositories.

If there are ethical or legal restrictions on sharing a de-identified data set, please explain them in detail (e.g., data contain potentially sensitive information, data are owned by a third-party organization, etc.) and who has imposed them (e.g., an ethics committee). Please also provide contact information for a data access committee, ethics committee, or other institutional body to which data requests may be sent. If data are owned by a third party, please indicate how others may request data access.

3. Your ethics statement should only appear in the Methods section of your manuscript. If your ethics statement is written in any section besides the Methods, please move it to the Methods section and delete it from any other section. Please ensure that your ethics statement is included in your manuscript, as the ethics statement entered into the online submission form will not be published alongside your manuscript.

4. If the reviewer comments include a recommendation to cite specific previously published works, please review and evaluate these publications to determine whether they are relevant and should be cited. There is no requirement to cite these works unless the editor has indicated otherwise.

5. Please review your reference list to ensure that it is complete and correct. If you have cited papers that have been retracted, please include the rationale for doing so in the manuscript text, or remove these references and replace them with relevant current references. Any changes to the reference list should be mentioned in the rebuttal letter that accompanies your revised manuscript. If you need to cite a retracted article, indicate the article’s retracted status in the References list and also include a citation and full reference for the retraction notice.

[Note: HTML markup is below. Please do not edit.]

Reviewers' comments:

Reviewer's Responses to Questions

**Comments to the Author**

1. Is the manuscript technically sound, and do the data support the conclusions?

Reviewer #1: Yes

Reviewer #2: No

Reviewer #3: Yes

Reviewer #4: Partly

**********

2. Has the statistical analysis been performed appropriately and rigorously?

Reviewer #1: Yes

Reviewer #2: No

Reviewer #3: Yes

Reviewer #4: Yes

**********

3. Have the authors made all data underlying the findings in their manuscript fully available?

The PLOS Data policy

Reviewer #1: Yes

Reviewer #2: Yes

Reviewer #3: Yes

Reviewer #4: Yes

**********

4. Is the manuscript presented in an intelligible fashion and written in standard English?

Reviewer #1: Yes

Reviewer #2: No

Reviewer #3: Yes

Reviewer #4: Yes

**********

Reviewer #1: 1. the title could be Incidence and predictors of mother-to-child transmission of HIV among HIV exposed infants in Tigray, Ethiopia: A retrospective cohort study (consider either public or general hospital)

2. in the abstract, the last paragraph of the background should be rephrased for clarity

3. correct grammar in lines 79 and 80

4. in line 101 1nd 102 it may be necessary to site some of the other studies

5. rephrase line 138

6. in the section of independent variables, father HIV status and Enrolment to ART an infant factor?

7. It might be necessary to site some of the resources from where the data extraction form was adapted from

8. What was the detailed information on the records and management of the infants obtained from key informants (health care providers) working at the PMTCT unit used for as indicated in lines 243 and 244?

9. In line 256, for clarity it maybe necessary to indicate the modifications done on the data extraction form after pre testing

10. From line 256, I am not sure how pre testing was used to later check for completeness and accuracy before both the data collectors and supervisors go to the field

11. Rephrase line 274 and 275 for clarity

12. Check spelling of last on table 3

13. Be consistent with the use of either British or American English

Reviewer #2: This is an important topic that deserves research/scrutiny but the manuscript and analysis have several significant flaws that make the findings uninterpretable. Key ones include:

- Although the study sample includes HEIs who registered in PMTCT (the majority at the age of 6 weeks), there is no mention of HEI loss to follow-up, proportion of HEIs who received recommended infant virologic testing, or HEIs who received the final test after breastfeeding cessation. Given the high rates of LTFU for HEIs, the very low coverage of EID in Ethiopia for the 6 week test (and even lower coverage for the 9 month virologic test), the denominator of HEIs used for the MTCT rate is inherently inaccurate and unreliable.

- In Table 3, there is no delineation of missing data for CD4 and viral load testing. This is not believable for any clinical setting (especially one that is a retrospective review of routine program data). Coverage of viral load testing in pregnant women in recent data was under 70% and casts doubt on the credibility of this and all other data tables presented.

- Missing data (clinical, laboratory, and engagement in care/retention) is critical for these types of programmatic analyses and is ignored throughout.

- There are numerous typographical and word errors throughout.

- Several pieces of epidemiologic data and references within the introduction are severely outdated (e.g., 2016-2018) despite availability of more recent estimates.

Reviewer #3: This tidy and convincing study aims to add to the data available on PMTCT in Ethiopia by addressing important variables that were not sufficiently addressed previously, and specifically determine the incidence and predictors of MTCT of HIV among HIV-exposed infants in selected public general hospitals of Tigray, Ethiopia. It was conducted in public General Hospitals of Tigray in early 2020 and covers HIV-exposed infants enrolled in PMTCT there. Data were collected primarily from hospital medical records, and health care providers at the PMTCT units of the hospitals.

Ethical clearance was obtained in 2020, and data collection instruments pre-tested.

A total of 444 (91.5%) mother–infant pairs were eligible and included in the final analysis. The study process is well-described and appears quite valid and thorough. Socio-demographical, clinical and obstetrical characteristics of the mothers and characteristics of HIV exposed infants was collected. Bivariate and Multivariable analysis of the data was conducted (see below).

The authors describe their findings and note differences and similarities with other such data from within Ethiopia and Africa and other parts of the world. They note that the study shows good progress on the elimination of MTCT of HIV among HIV exposed infants in the study area, but still, it is higher than the benchmark of the health sector transformation plan of Ethiopia which was targeted to be 0% by 2020.

The study is by and large well-written, though some slight editing is needed especially in the abstract and introduction, and well-referenced.

Unfortunately, the study is rather out-of-date. It would appear that the study was started before the war in Tigray started, and could not be concluded until well after it ended. Many of the references are from 2015-18; the data for PMTCT and HIV globally, for example, could easily have been updated to 2024 from UNICEF, UNAIDS or WHO recent websites. A simple online search for recent articles on PMTCT in Ethiopia, even Tigray itself, finds articles from the BMJ (May 2026), PubMed (May 2025), the Journal of the International Association of Providers of AIDS Care (November 2021), Frontiers of Reproductive Health (November 2024), BMC Pediatrics (2024), HIV AIDS (Aukland) (2021), Italian Journal of Paediatrics (2021), etc. Some of these are cited as annex.

Thus, unless the authors specifically state that this is a historical snap-shot from 2020, the article has little relevance to today. I will leave it to the authors to compare their 2020 findings to more recent findings; and to explain the time-lag. This will give the article much more interest, to show what progress has (or has not) been made since 2020, in Ethiopia, particularly within the context of the conflict in Tigray.

Back within the present article, I am not qualified properly to assess the validity or quality of the statistical analysis done; though it looks all right to me.

Annex:

Facha W, Tadesse T, Wolka E, Astatkie A. Magnitude and risk factors of mother-to-child transmission of HIV among HIV-exposed infants after Option B+ implementation in Ethiopia: a systematic review and meta-analysis. AIDS Res Ther. 2024 Jun 7;21(1):39. doi: 10.1186/s12981-024-00623-6. PMID: 38849895; PMCID: PMC11157738.

Gebremedhin H, Gebremeskel F, Gebreegziabiher G, Atsbaha AH, Hailesilase GG. Determinants of HIV infection among infants born to HIV positive women receiving option B + prevention of mother to child transmission of HIV in Tigray, north Ethiopia: a case control study. AIDS Res Ther. 2025 May 30;22(1):55. doi: 10.1186/s12981-025-00755-3. PMID: 40448184; PMCID: PMC12125716.

Tilaye BA, Hailemeskel HS, Baye FD, Hailu MK, Mekonnen GB, Arage G, Birhane BM, Tibebu NS, Moges N. Incidence of mother-to-child transmission of HIV and predictors of positivity among HIV exposed infants in South Gondar public hospitals, Northwest Ethiopia: competing risk regression model. BMC Pediatr. 2024 Sep 20;24(1):597. doi: 10.1186/s12887-024-05061-x. PMID: 39304894; PMCID: PMC11414292.

Degavi G, Safayi BL, Adola SG, Demisse B, Utura T, Gemeda U, Kelna Edwin SE, Demissie F. A Retrospective Study of Incidence and Predictors on Mother-to-Child Transmission of HIV among HIV-Exposed Infants in West Guji Zone, Southern Ethiopia. AIDS Res Treat. 2022 Feb 23;2022:2906490. doi: 10.1155/2022/2906490. PMID: 35251712; PMCID: PMC8890858.

Alemu A, Molla W, Yinges K, Mihret MS. Determinants of HIV infection among children born to HIV positive mothers on prevention of mother to child transmission program at referral hospitals in west Amhara, Ethiopia; case control study. Ital J Pediatr. 2022 Feb 3;48(1):17. doi: 10.1186/s13052-022-01220-x. Erratum in: Ital J Pediatr. 2022 Feb 24;48(1):34. doi: 10.1186/s13052-022-01230-9. PMID: 35115031; PMCID: PMC8812050.

Tadewos K, Adimasu M, Tachbele E. Mother-to-Child Transmission of HIV and Associated Factors Among Exposed Infants in Pastoralist Health Facilities, South Omo Zone, Ethiopia, 2020 - A Retrospective Cross-Sectional Study. HIV AIDS (Auckl). 2021 Dec 9;13:1015-1023. doi: 10.2147/HIV.S332904. PMID: 34916853; PMCID: PMC8668224.

Reviewer #4: Overall assessment

This manuscript addresses an important public health issue, mother-to-child transmission (MTCT) of HIV, in a high-burden, resource-limited setting. The study contributes regionally relevant data from multiple hospitals in Tigray, Ethiopia, and attempts to incorporate underexplored predictors such as paternal ART enrollment. The reported MTCT incidence (3.8%) reflects progress toward elimination targets but highlights persistent gaps.

While the topic is relevant and potentially publishable, the manuscript requires substantial revisions in methodological clarity, analytical rigor, and language quality before it meets the standards expected by PLOS journal.

Major strengths

• Multicentre design enhances generalizability compared to single-site studies common in Ethiopia. This was well done.

• Relatively large sample size (n=444) which improves statistical power.

• Inclusion of novel predictors (e.g., paternal ART enrollment) strengthens the contribution to the literature.

• Use of routine PMTCT program data enhances real-world applicability.

• The estimated MTCT rate (3.8%) is contextualized against national and global benchmarks.

Major concerns

1. Conceptual and analytical issues

• The study is described as a retrospective cohort, yet the analysis relies solely on binary logistic regression, with no time-to-event consideration.

→ This raises concerns about whether "incidence" is appropriately measured (appears closer to cumulative proportion rather than true incidence).

• Confounding control is limited

o Important variables (e.g., maternal viral load, despite being mentioned as a gap in prior studies) are not clearly integrated into the final model.

o Wide confidence intervals (e.g., AOR up to 44.7) suggest model instability or possible overfitting.

• The inclusion criterion requiring confirmed outcomes may introduce selection bias, excluding infants lost to follow-up.

2. Methodological Clarity

• The sampling procedure lacks clarity:

o Justification for selecting 40% of hospitals is not provided.

• The definition of exposure period and follow-up time is inadequate:

o No clear indication of person-time or censoring.

• Handling of missing data is not described.

3. Interpretation of Findings

• The conclusion that MTCT is “still high” is overstated given that 3.8% is close to or within elimination targets (<5% breastfeeding settings).

• Some interpretations are overly speculative, particularly regarding paternal ART mechanisms without direct measurement.

4. Language and Scientific Writing

• The manuscript requires substantial language editing:

o Frequent grammatical errors

o Inconsistent tense usage

• Some statements lack precision (e.g., “this study was identified”).

Recommendations for improvement

1. Clarify study design and analysis

o Justify the use of logistic regression vs. survival analysis.

o Clearly define incidence measurement.

2. Strengthen modelling

o Address confounding systematically.

o Report model diagnostics more robustly.

3. Improve methodological transparency

o Clearly describe sampling rationale.

o Address missing data and potential biases.

4. Refine interpretation

o Align conclusions with actual magnitude of findings.

o Avoid overgeneralization.

5. Revise language thoroughly

o Professional editing is strongly recommended.

Final recommendation

Revision Required

The manuscript has merit and contributes relevant regional evidence; however, improvements in methodological rigor, clarity, and scientific writing are necessary before it can be considered for publication.

**********

what does this mean?). If published, this will include your full peer review and any attached files.

If you choose “no”, your identity will remain anonymous but your review may still be made public.

**Do you want your identity to be public for this peer review?**  For information about this choice, including consent withdrawal, please see our Privacy Policy

Reviewer #1: No

Reviewer #2: No

Reviewer #3: **Yes:**  Peter Godwin

Reviewer #4: **Yes:**  Wilbroda Hlolisile Chiya

**********

[NOTE: If reviewer comments were submitted as an attachment file, they will be attached to this email and accessible via the submission site. Please log into your account, locate the manuscript record, and check for the action link "View Attachments". If this link does not appear, there are no attachment files.]

To ensure your figures meet our technical requirements, please review our figure guidelines: https://journals.plos.org/plosone/s/figures

You may also use PLOS’s free figure tool, NAAS, to help you prepare publication quality figures: https://journals.plos.org/plosone/s/figures#loc-tools-for-figure-preparation.

NAAS will assess whether your figures meet our technical requirements by comparing each figure against our figure specifications.

# Author comment: Author response to Decision Letter 1

**DOI:** 10.1371/journal.pone.0358878.r002

5 Sep 2026

We have respond to each reviewers comments point by point.

Attachment Submitted filename: Response to Reviewers.docx

# Editor report: Decision Letter 1

**DOI:** 10.1371/journal.pone.0358878.r003

**Authors:** David Ikwuka

8 Sep 2026

Incidence and predictors of mother-to-child transmission of HIV among HIV exposed infants attending in public General Hospitals of Tigray, Ethiopia, 2020: retrospective cohort study

PONE-D-26-12519R1

Dear Dr. Gerezgiher,

We’re pleased to inform you that your manuscript has been judged scientifically suitable for publication and will be formally accepted for publication once it meets all outstanding technical requirements.

Within one week, you’ll receive an e-mail detailing the required amendments. When these have been addressed, you’ll receive a formal acceptance letter and your manuscript will be scheduled for publication.

An invoice will be generated when your article is formally accepted. Please note, if your institution has a publishing partnership with PLOS and your article meets the relevant criteria, all or part of your publication costs will be covered. Please make sure your user information is up-to-date by logging into Editorial Manager at Editorial Manager® and clicking the ‘Update My Information' link at the top of the page. For questions related to billing, please contact billing support.

If your institution or institutions have a press office, please notify them about your upcoming paper to help maximize its impact. If they’ll be preparing press materials, please inform our press team as soon as possible -- no later than 48 hours after receiving the formal acceptance. Your manuscript will remain under strict press embargo until 2 pm Eastern Time on the date of publication. For more information, please contact onepress@plos.org.

Kind regards,

David Chibuike Ikwuka, Ph.D.

Academic Editor

PLOS One

Additional Editor Comments (optional):

Reviewers' comments:

# Editor report: Acceptance letter

**DOI:** 10.1371/journal.pone.0358878.r004

**Authors:** David Ikwuka

PONE-D-26-12519R1

PLOS One

Dear Dr. Gerezgiher,

I'm pleased to inform you that your manuscript has been deemed suitable for publication in PLOS One. Congratulations! Your manuscript is now being handed over to our production team.

At this stage, our production department will prepare your paper for publication. This includes ensuring the following:

* All references, tables, and figures are properly cited

* All relevant supporting information is included in the manuscript submission,

* There are no issues that prevent the paper from being properly typeset

You will receive further instructions from the production team, including instructions on how to review your proof when it is ready. Please keep in mind that we are working through a large volume of accepted articles, so please give us a few days to review your paper and let you know the next and final steps.

Lastly, if your institution or institutions have a press office, please let them know about your upcoming paper now to help maximize its impact. If they'll be preparing press materials, please inform our press team within the next 48 hours. Your manuscript will remain under strict press embargo until 2 pm Eastern Time on the date of publication. For more information, please contact onepress@plos.org.

You will receive an invoice from PLOS for your publication fee after your manuscript has reached the completed accept phase. If you receive an email requesting payment before acceptance or for any other service, this may be a phishing scheme. Learn how to identify phishing emails and protect your accounts at https://explore.plos.org/phishing.

If we can help with anything else, please email us at customercare@plos.org.

Thank you for submitting your work to PLOS One and supporting open access.

Kind regards,

PLOS One Editorial Office Staff

on behalf of

Dr David Chibuike Ikwuka

Academic Editor

PLOS One
